# Public health round-up

**DOI:** 10.2471/BLT.25.010625

**Published:** 2025-06-01

**People in Gaza starving as aid blockade continues**

The risk of famine in Gaza is increasing with the deliberate withholding of humanitarian aid, including food, in the ongoing blockade. The entire 2.1 million population of Gaza is facing prolonged food shortages, with nearly half a million people in a catastrophic situation of hunger, acute malnutrition, starvation, illness and death.

**Figure:**
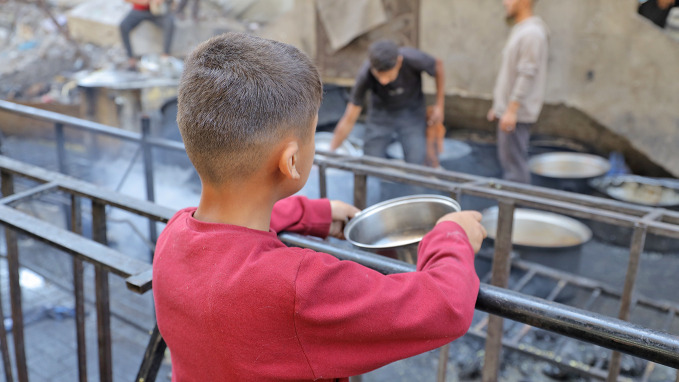
WHO

## Pandemic Agreement

Member States of the World Health Organization (WHO) have formally adopted by consensus the world’s first Pandemic Agreement during the 78th World Health Assembly. This landmark decision follows over three years of negotiations sparked by the coronavirus disease 2019 (COVID-19) pandemic and aims to strengthen global preparedness and equity in future health crises.

“This historic Agreement is a victory for public health, science, and multilateral action,” said WHO Director-General Tedros Adhanom Ghebreyesus. “It ensures we are better equipped to protect the world from future pandemics.”

The Agreement was adopted after a committee vote (124 in favour, 0 objections, 11 abstentions) and emphasizes equitable access to vaccines, therapeutics and diagnostics. It also affirms national sovereignty, stating WHO cannot mandate domestic laws or policies, including lockdowns or vaccine mandates.

The resolution on the WHO Pandemic Agreement adopted by the World Health Assembly sets out steps to prepare for the accord’s implementation. It includes launching a process to draft and negotiate a Pathogen Access and Benefit Sharing system (PABS) through an Intergovernmental Working Group. The result of this process will be considered at next year’s Assembly.

https://bit.ly/43pogCI

## Immunization for respiratory syncytial virus

WHO has released its first position paper on immunization products to protect infants against respiratory syncytial virus (RSV), a major cause of acute lower respiratory infections in young children. Globally, RSV is responsible for around 100 000 deaths and 3.6 million hospitalizations annually among children under five, with infants under six months of age most at risk, especially in low- and middle-income countries.

Published in the *Weekly Epidemiological Record*, the paper outlines recommendations for two key interventions: a maternal vaccine (RSVpreF) and a long-acting monoclonal antibody (nirsevimab). The maternal vaccine, approved by WHO in March 2025, is recommended during the third trimester to transfer protective antibodies to the baby before birth. Nirsevimab, administered as a single injection shortly after birth, provides protection for at least five months.

Both products were endorsed by WHO’s Strategic Advisory Group of Experts on Immunization in September 2024. Countries are encouraged to select the most feasible option based on their health-care infrastructure, cost–effectiveness and coverage potential. These tools represent a major advance in preventing severe RSV illness and deaths in infants worldwide.

https://bit.ly/4dOU2MY

## Ban on flavoured tobacco 

On World No Tobacco Day, WHO has issued a call for urgent action: ban all flavoured tobacco and nicotine products, including cigarettes, hookahs, e-cigarettes and pouches, to curb youth addiction and protect public health.

Flavours like menthol, cotton candy and bubble gum disguise the dangers of tobacco and nicotine, making harmful products more attractive to young people. These flavours not only increase the risk of addiction but are also linked to serious lung diseases and reduced quitting success.

“Flavours are fuelling a new wave of addiction and undermining decades of progress,” said WHO Director-General Tedros Adhanom Ghebreyesus. The new WHO publication, *Flavour accessories in tobacco products enhance attractiveness and appeal*, reveals how industry tactics, like capsule filters and click-on flavour drops, are designed to sidestep regulations and entice new users.

Although over 50 countries have banned flavoured tobacco and more than 40 restrict e-cigarette sales, WHO stresses that flavour accessories remain largely unregulated. WHO calls on all nations to follow countries like Belgium, Denmark and Lithuania in tightening restrictions.

https://bit.ly/43KKmi3

## Disease surveillance in Africa

WHO, the Africa Centres for Disease Control and Prevention (Africa CDC) and the Robert Koch Institute (RKI), with support from the governments of Canada and the United Kingdom of Great Britain and Northern Ireland, are expanding the Health Security Partnership to Strengthen Disease Surveillance in Africa (HSPA) to seven countries. Initially launched in 2023 in six countries (Gambia, Mali, Morocco, Namibia, South Africa and Tunisia), the initiative now includes Rwanda in its second phase (2025–2028).

Africa faces more disease outbreaks than any other region, making strong surveillance systems critical. HSPA enhances countries’ capacity to detect and respond to public health threats, natural, accidental, or deliberate, through a collaborative surveillance approach that links health and security sectors.

Aligned with global health security efforts like the Global Partnership and the Signature Initiative to Mitigate Biological Threats in Africa (SIMBA), HSPA supports bio-risk management, genomic and epidemic surveillance, and early warning systems. This is achieved through technical assistance, training and the co-development of national implementation roadmaps.

WHO, Africa CDC and partners emphasize the importance of regional coordination and national leadership. “This initiative strengthens health security through shared action and sustainable systems,” said Dr Chikwe Ihekweazu, WHO Regional Director for Africa *ad interim*. 

https://bit.ly/3FDIbog

## Safer and healthier mobility

WHO has launched a new toolkit to help governments make active mobility safer and more accessible.

Each year, nearly 1.2 million people die in road crashes, over a quarter of these people are killed while walking or cycling. Despite the clear health and environmental benefits of active travel, fewer than one-third of countries have national policies promoting walking and cycling. Notably, only 0.2% of roads worldwide feature dedicated cycle lanes and many communities lack sidewalks or safe crossings. 

WHO’s *Promoting walking and cycling: a toolkit of policy options* offers practical, evidence-based guidance for policy-makers, urban planners and advocates. It calls for integrating active mobility into broader policies, investing in safe infrastructure, enforcing speed limits aligned with best practices, promoting public awareness and using financial incentives to encourage walking and cycling.

“It is urgent to make, what should be our most natural means of transport, safer. This is paramount for road safety, but also health, equity and climate,” said Dr Etienne Krug, director of the WHO Department for the Social Determinants of Health. 

https://bit.ly/3ZJzOyi

https://bit.ly/3FDUgKc

## Impact of inequities on health

A new global WHO report reveals that social and economic inequities like poor housing, limited education, and lack of job opportunities, can shorten healthy life expectancy by decades, even in high-income countries. The *World report on social determinants of health equity* highlights that where people live, grow, work and age influences health more than genetics or access to care.

People born in countries with the lowest life expectancy live, on average, 33 years less than those in countries with the highest. Within countries, health follows a clear social gradient: the poorer and more disadvantaged a community, the worse its health outcomes. These disparities are especially stark among marginalized populations, such as Indigenous peoples, who often face shorter life expectancies regardless of national wealth.

While maternal mortality has globally declined 40% since 2000, 94% of maternal deaths still occur in low- and lower-middle-income countries. Disadvantaged women face higher risks in pregnancy and childbirth, and racial and gender inequalities persist even in wealthy countries.

The report calls for urgent, cross-sector action to tackle income inequality, discrimination, and climate-related disruptions. “Our world is an unequal one,” said WHO Director-General Tedros Adhanom Ghebreyesus. “But change for the better is possible.” 

https://bit.ly/3T3NCjk

## New guidance to end medicalized female genital mutilation

WHO has issued new guidelines to curb the rising trend of medicalized female genital mutilation (FGM) and strengthen support for survivors. Despite global efforts to end FGM, an estimated 52 million girls and women (nearly 1 in 4 cases) have undergone the procedure at the hands of health workers, raising serious concerns about the role of the health sector.

The *WHO Guideline on the prevention of female genital mutilation and clinical management of complications*, provides evidence-based recommendations for health systems, governments and communities. It calls for strict codes of conduct to ban health workers from performing FGM and urges their active engagement in prevention through education, counselling and advocacy.

“FGM is a severe violation of girls’ rights and critically endangers their health,” said Dr Pascale Allotey, WHO director for Sexual and Reproductive Health and Research. “Health workers must be agents for change, not perpetrators of this harmful practice.”

The guideline also outlines clinical care strategies to address both immediate and long-term health effects of FGM, including mental health support and surgical interventions. It stresses the importance of community engagement, including with men and boys, to shift norms and reduce prevalence.

“Engaging doctors, nurses and midwives should be a key element in FGM prevention and response,” said Christina Pallitto, WHO scientist and lead author.

https://bit.ly/4kKKogv

https://bit.ly/3FKibHP

**Cover photo**

Children walk and run in the streets of Chajul, Quiche, Guatemala.

**Figure:**
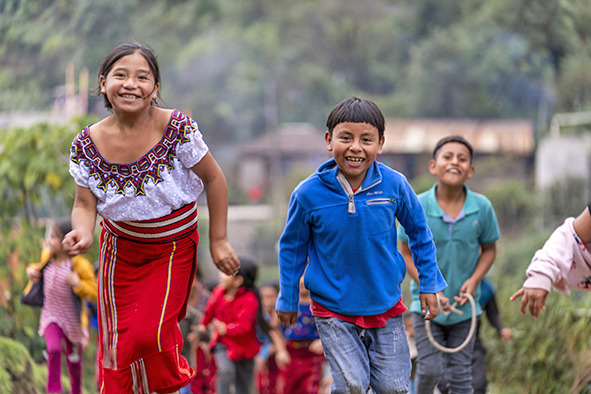
(UNICEF/UNI465672/Flores)

**Looking ahead**

14 June. World Blood Donor Day 2025, Give blood, give hope: together we save lives. https://bit.ly/4dKZ3WX

24–26 June. Fifth WHO Forum on Alcohol, Drugs and Addictive Behaviours. https://bit.ly/3ZcWpmP

